# Enhanced radiosensitivity of head and neck cancer cells to proton therapy via hyperthermia-induced homologous recombination deficiency

**DOI:** 10.1016/j.ctro.2024.100898

**Published:** 2024-12-04

**Authors:** Tim Heemskerk, Gerarda van de Kamp, Marta Rovituso, Roland Kanaar, Jeroen Essers

**Affiliations:** a Department of Molecular Genetics, Oncode Institute, Erasmus MC Cancer Institute, Erasmus University Medical Center, Rotterdam, Netherlands (the); b R&D Department, HollandPTC, Delft, Netherlands (the); c Department of Vascular Surgery, Erasmus University Medical Center, Rotterdam, Netherlands (the); d Department of Radiotherapy, Erasmus MC Cancer Institute, Erasmus University Medical Center, Rotterdam, Netherlands (the)

**Keywords:** DNA breaks, Double-stranded, Proton therapy, Rad51 recombinase, Squamous cell carcinoma of head and neck, Hyperthermia, Homologous recombination, BRCA2 protein

## Abstract

**Background and purpose:**

Radiotherapy induces tumor cell killing by generating DNA double strand breaks (DSBs). The effectiveness of radiotherapy is significantly influenced by the repair of DSBs, which counteracts this lethal effect. Current investigations are focused on determining whether non-homologous end joining (NHEJ) or homologous recombination is the predominant repair pathway following proton and photon radiation.

**Materials and methods:**

In this study, we examined the response of FaDu cells, a head and neck squamous cell carcinoma model, to spread-out Bragg peak (SOBP) proton and photon radiation combined with mild hyperthermia (42 °C for one hour) to induce homologous recombination deficiency or NHEJ inhibition by AZD7648.

**Results:**

Hyperthermia resulted in stronger radiosensitization after proton radiation (SR = 1.53) compared to photon radiation (SR = 1.32). Conversely, NHEJ inhibition did not produce a significant differential effect between photon and proton radiation. This indicates a greater reliance on homologous recombination following proton radiation compared to photon radiation. We found that the number of DSBs formed after photon versus proton irradiation is comparable. Interestingly, the homologous recombination protein Rad51 accumulated more frequently at DSBs following proton irradiation than photon irradiation.

**Conclusions:**

These findings support the hypothesis that cells rely more on homologous recombination to repair proton-induced DNA damage compared to photon-induced DNA damage. As clinically applied hyperthermia enhances the therapeutic effect of photon irradiation by, among other factors, inducing homologous recombination deficiency, our results suggests that hyperthermia could be more effective in combination with proton irradiation than photon irradiation.

## Highlights

• Hyperthermia induces stronger radiosensitization to proton than to photon radiation.
• Rad51 accumulates more frequently at double strand breaks after proton radiation.
• HNSCC relies more on HR to repair damage induced by proton than photon radiation.

## Introduction

Interest in proton radiotherapy for tumor treatment has increased over the past decades, due to its superior spatial dose distribution in tissue compared to conventional radiotherapy. Photons deposit the maximum dose at the entrance of the tissue, with the dose gradually declining thereafter. In contrast, protons deposit a relatively low dose at the entrance, while at the end of their range the dose sharply rises, forming a so-called Bragg peak [1]. This allows for the delivery of the major dose to the tumor while sparing the surrounding healthy tissue. Furthermore, the linear energy transfer (LET), the amount of energy deposited per unit length, also changes through the Bragg peak. While protons are considered to be low LET radiation, like photons, their LET increases sharply towards the end of the Brag peak [2]. This higher LET means denser ionization events, which can result in more complex DNA damage, consisting of multiple types DNA damage localized over a short distance [2], [3].

Radiotherapy induces tumor cell killing by producing DNA double strand breaks (DSBs). The repair of these DSBs is a critical determinant of the effectiveness of radiotherapy, as it counteracts the lethal effect of the breaks. DSBs can be repaired by three major DSB repair pathways: non-homologous end joining (NHEJ), theta-mediated end joining and homologous recombination [4]. Whereas NHEJ and theta-mediated end joining are prone to errors, homologous recombination uses a homologous template to repair the break in an error-free fashion. At present, the importance of each pathway to repair proton-induced DSBs is still debated [4], [5]. One hypothesis is that protons, especially in the Bragg peak, induce more complex DNA damage. The combination of multiple types of lesions close together could have an influence on the used DSB repair pathway [2], [4]. For instance, long single-stranded DNA tails or single-strand DNA gaps near the DSB site can block Ku70/80 binding, leading to resection of the break ends [6]. Some studies indeed show a stronger dependence on homologous recombination for repair of proton-induced DNA damage compared to photon-induced DNA damage [7], [8]. Additional studies show that the effect is specific for high LET protons [9], [10]. However, there are also studies that have not been able to show a stronger dependence on homologous recombination after proton radiation [11], [12]. Loss of NHEJ often does lead to a strong radiosensitization, but this is mostly independent of the radiation type [7], [8], [9], [11], [12], [13].

Hyperthermia treatment has been combined with radiotherapy as a radiosensitizer [14], [15]. Mild hyperthermia (42 °C for 1 h) induces BRCA2 degradation, an essential protein for DSB repair by homologous recombination [16]. Without BRCA2, Rad51 cannot be loaded onto resected DNA, preventing the critical homology search [4]. This hyperthermia-mediated BRCA2 degradation sensitizes cells to photon radiation [17]. In this study, we investigated the sensitization of cells to proton irradiation by hyperthermia. We hypothesized that hyperthermia would enhance cellular sensitivity to proton irradiation more than to photon irradiation, based on the rationale that cells would depend more on homologous recombination for repairing the complex proton-induced DNA breaks [2].

We investigated the response of a head and neck squamous cell carcinoma cell line, FaDu, to photon and proton irradiation with or without hyperthermia treatment. We also examined radiosensitization induced by NHEJ inhibition. The dependence on homologous recombination was further investigated by studying the accumulation of DNA repair proteins 53BP1 and Rad51 after a dose gradient of proton and photon radiation. 53BP1 accumulates at DSBs into distinct foci at the damage site, which allows for its utilization as a marker to visualize DSBs [18]. Rad51 is a key player in homologous recombination and its accumulation in foci can be used as a marker for utilization of homologous recombination [4], [19]. Together these experiments provide more insight in DSB repair mechanisms of proton-induced DNA damage response, enabling rational design of combination therapies to improve the effectiveness of proton therapy.

## Materials and methods

### Cell culture

FaDu (HTB-43, ATCC) were cultured in a 1:1 mixture of Dulbecco's Modified Eagle Medium (4.5 g/L glucose, with l-glutamine) and Ham’s F-10, supplemented with 10 % fetal calf serum and 1 % penicillin/streptomycin, and were maintained in a humidified incubator with 37 °C and 5 % CO_2_.

### X-ray and proton irradiation

X-ray irradiations were performed using the RS320 (Xstrahl Live Sciences), a self-contained cabinet, with a dose rate of 1.6 Gy/min and working voltage of 195 kV and 10 mA.

**Fig. 1:**
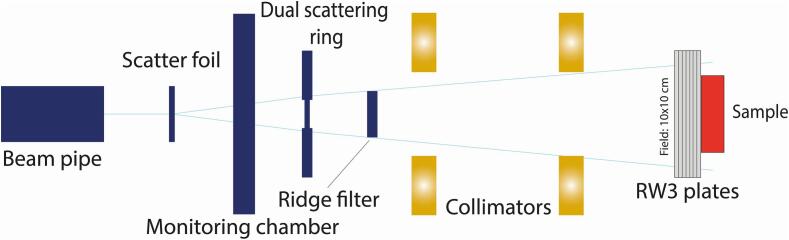
HollandPTC R&D beam line. Schematic representation of the HollandPTC R&D beam line components. The proton beam leaves the beam pipe and is diverted by the scatter foil and dual scattering ring. The ridge filter changes the Bragg curve into a SOBP. Using the collimators the beam is shaped to a 10 × 10 cm field. Finally, the protons hit the RW3 plates to position the sample in the middle of the SOBP.

Proton irradiations were performed at the research beam line of the Holland Proton Therapy Center (Delft, the Netherlands). This is equipped with a passive scattering beam as described in Rovituso et al. [20] (Fig. 1). Samples were irradiated in the middle of the spread-out Bragg peak (SOBP) (3 cm) with a beam energy of 150 MeV. For all clonogenic survivals, a setup with a 10 × 10 cm field size was used with a dose rate of 1.4 Gy/min. For the irradiation of coverslips for the foci experiments, a setup with an 8 × 8 cm field size was used with a dose rate of 5.7 Gy/min. All doses presented are the physical dose and not corrected for the relative biological effectiveness of each radiation type.

### Clonogenic survival assay – irradiation + hyperthermia

Hyperthermia was applied by placing the samples in an incubator at 42 °C. A heating period of 12 min was used to heat the samples from 37 °C to 42 °C, followed by a hyperthermia period of 1 h at 42 °C. Temperature of the samples was monitored with thermocouples to assure the desired temperature was reached.

One day prior to the irradiation, 1.5*10^6^ FaDu cells were seeded in T25 flasks. The cells were allowed to attach overnight and were treated with either normothermia (37 °C) or hyperthermia (42 °C) for 1 h followed directly by irradiation with either X-ray or protons (0, 2, 4 and 6 Gy). Afterwards, the cells were trypsinized, counted and seeded in triplicates in 6 cm dishes. The amount of cells seeded was adjusted based on the dose given, with 400, 800, 1600, 3500 cells per dish for the doses 0, 2, 4, 6 Gy, respectively. Additionally, for the hyperthermia treated samples, cells were also seeded in double the concentration for 4 and 6 Gy samples to compensate for the increased cell killing of the hyperthermia treatment. Colonies were allowed to form for 14 days, after which they were fixed and stained in Coomassie Blue staining solution (50 % methanol, 7 % acetic acid, 43 % demi water, 0.1 % brilliant blue R). Colonies were counted with the GelCount colony counter (Oxford optronic). To visualize the survival curves, the data of three independent experiments were pooled and a linear-quadratic survival curve was fitted to the data points, utilizing weighted least squares regression in GraphPad Prism 9. To assess the relative sensitivity of cell survival curves in comparison to each other, a linear quadratic survival curve was fitted to the clonogenic survival data of each independent experiment, utilizing weighted least squares regression in GraphPad Prism 9. From the fitted curve, the dose resulting in 10 % survival (D10) was derived and the sensitization ratio (SR) was calculated, defined as the D10 of monotherapy treated cells divided by the D10 of the combination treated cells. The obtained D10 and SR values of three independent experiments were pooled and from this the mean was determined. Significance was calculated in GraphPad Prism 9, using Sidáks multiple comparisons test for the D10 values and an unpaired *t*-test for the significance of the SR.

### Clonogenic survival assay – irradiation + AZD7648

NHEJ was inactivated by pharmacological inhibition of DNA-PKcs using AZD7648. The concentration and timing of the inhibitor was optimized for the FaDu cells to induce a strong inhibition of DNA-PKcs autophosphorylation on Ser2056, while having limited toxicity.

One day prior to the irradiation, 1.5*10^6^ FaDu cells were seeded in T25 flasks. The cells were allowed to attach overnight. One hour prior to irradiation, 0.1 µM AZD7648 was added to the flasks, after which the cells were irradiated with either X-ray or protons (0, 2, 4, 6 Gy). The cells were trypsinized, counted and seeded in triplicates in 6 cm dishes in the presence of 0.1 µM AZD7648. The amount of cells seeded was adjusted based on the dose given, with 400, 800, 1600, 3500 cells per dish for the doses 0, 2, 4, 6 Gy respectively. Additionally, for the inhibitor treated samples, cells were also seeded in 4 times the concentration for 4 and 6 Gy samples to compensate for the increased cell killing of the inhibitor treatment. Twenty four hours after irradiation the inhibitor was washed away and colony formation was allowed for 13 additional days. Staining and quantification of the colony formation was performed as described above.

### Immunofluorescent staining

**Fig. 2:**
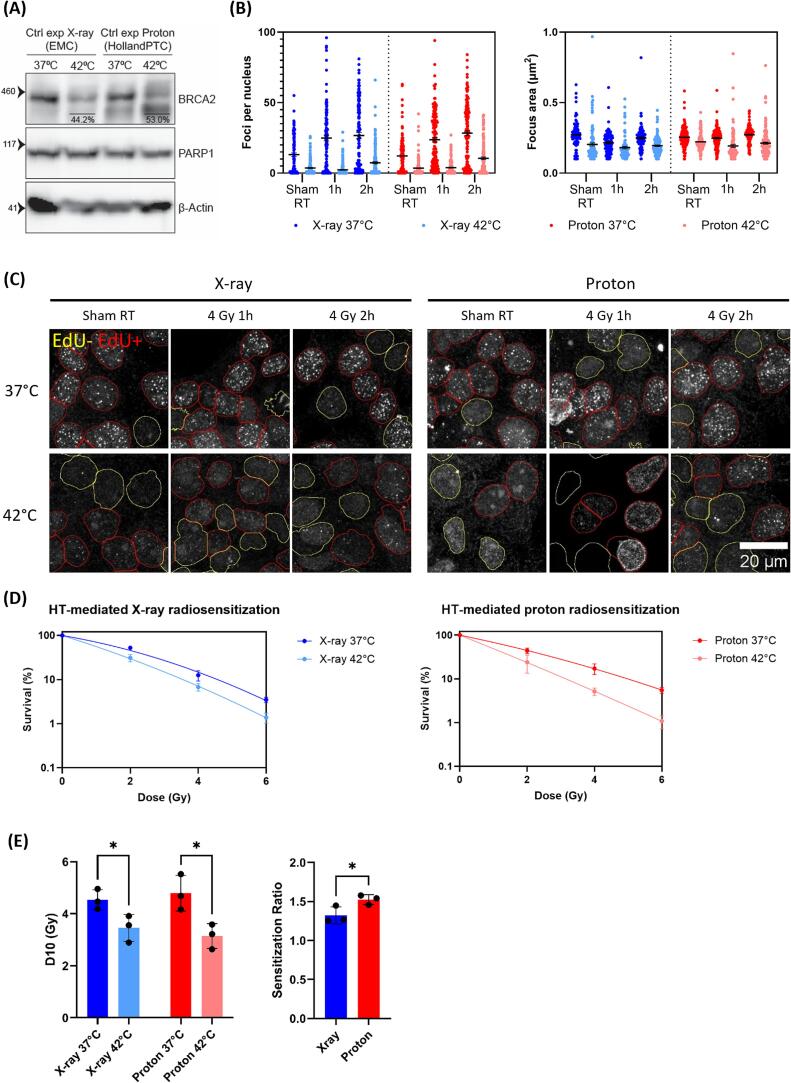
Hyperthermia-mediated radiosensitization. (A) Western blot to confirm BRCA2 degradation after HT treatment. PARP1 was used as a loading control. The percentage in the hyperthermia treated lanes indicate the remaining percentage of BRCA2 compared to the normothermia treated control. (B) Quantification of Rad51 foci per nucleus (left) and focus area (right) after irradiation with X-ray or protons combined with HT treatment. Bars represent mean and standard error of the mean. (C) Representative images of Rad51 foci after irradiation with either X-rays or protons combined with HT treatment. (D) Clonogenic survival after combination treatment of either X-ray or protons with hyperthermia treatment. Error bars represent standard deviation. (E) D10 (left) and SR (right) values obtained from the clonogenic survival after combination treatment of either X-ray or protons with hyperthermia. Each black dot represents an independent experiment. Error bars represent standard deviation.

One day prior to the irradiation, cells were seeded on 18 mm coverslips in 6-well plates at a density of 500.000 cells/well and were allowed to attach overnight. The coverslips were transferred to a 12-well plate. The coverslips for proton irradiation were attached to the well using PNIPAAm-PEG 3D thermoreversible hydrogel in cell culture medium (MBG-PMW20-1001, Mebiol Gel). The wells were filled completely with medium and sealed with Microseal B seals (MSB1001, Bio-Rad). The samples were irradiated with the indicated dose and fixed after 1 or 2 h (Fig. 2b) or 4 h for the dose gradient experiment. Forty five minutes prior to fixation, 30 µM 5-Ethynyl-2′-deoxyuridine (EdU) was added to allow EdU incorporation by replicating cells. At the indicated time points, cells were washed with cold phosphate buffered saline (PBS), incubated with ice-cold 0.5 % Triton X-100 extraction buffer (0.5 % Triton X-100, 20 mM HEPES-KOH (pH 7.9), 50 mM NaCl, 3 mM MgCl_2_, 300 mM sucrose) for 1 min, washed with cold PBS and fixed with 4 % paraformaldehyde (PFA) in PBS for 15 min at room temperature.

Cells were washed twice with 3 % BSA in PBS, permeabilized for 20 min with 0.5 % Triton X-100 in PBS and washed twice again with 3 % BSA in PBS. The cells were incubated for 30 min at room temperature with Click-iT reaction cocktail to label EdU with Atto488 or Atto647N (ATTO-TEC). The cells were washed once with 3 % BSA in PBS and once with PBS. Cells were washed twice with 0.1 % Triton and subsequently blocked with PBS + buffer (5 mg Bovine Serum Albumin (BSA) and 1.5 mg glycine/mL PBS). Primary antibodies (Rad51, rabbit, in house, 1:10000; 53BP1, mouse, Millipore, clone BP13, 1:1000) were diluted in PBS + buffer. Cells were incubated with primary antibodies overnight at 4 °C. Cells were permeabilized with 0.1 % Triton and washed with PBS + buffer. Secondary antibodies (anti-rabbit Alexa594, anti-mouse Alexa488, Life Technologies) were diluted in PBS + buffer (1:1000). Cells were incubated in the dark for 1 h at room temperature. Subsequently, the coverslips were mounted on microscope slides using Antifade mounting medium with DAPI (Vectashield). The coverslips were sealed with nail polish to prevent the samples from drying out.

### Microscopy

To visualize immunofluorescence in cells, a Leica STELLARIS 5 confocal microscope was employed. The following laser lines were used: DAPI (405 laser), Atto488 (488 laser), Alexa 594 (561 laser). For each sample, 5 Z-stack images were captured using a 40× objective. Subsequently, z-projections were generated and nuclear area, mean and integrated density of the DAPI signal, as well as EdU intensity, were measured for each nucleus. Additionally, Rad51 foci were counted, and their area and intensity were analyzed for each nucleus. This was accomplished using homemade ImageJ scripts. In short, cell nuclei were segmented based on the DAPI signal and the nuclear area and integrated density of the DAPI signal were quantified using the measurement function within ImageJ. For the identification of foci within the segmented nuclei, individual segmentation masks were created for each nucleus. Segmentation masks of the foci were generated using thresholds based on the mean + factor* standard deviation of the Rad51 signal [21]. The number, area and intensity of the segmented foci were then measured using the measurement function within ImageJ. A simple linear regression was fitted to the number of foci per nucleus data of the dose gradient experiment and it was determined if the slopes are significantly different using GraphPad Prism 9. A curve was not fitted for the focus size, but the difference between each dose was tested for significance using Sidáks multiple comparisons test using GraphPad Prims 9. The symbols have the following meaning: ns p > 0.05, * p ≤ 0.05, ** p ≤ 0.01, *** p ≤ 0.001, **** p ≤ 0.0001.

### Immunoblotting

To confirm BRCA2 degradation, cell lysates were made after hyperthermia treatment. One day prior to the hyperthermia treatment 2*10^6^ cells were seeded in 10 cm dishes. The next day the dishes were treated with hyperthermia for 1 h. Directly after, the cells were kept on ice and washed twice with ice-cold PBS, scraped from the dish and incubated in Laëmmli buffer (4 % SDS, 20 % glycerol, 120 mM Tris) for 5 min at 95 °C to lyse the cells. The samples were sheared with a syringe to reduce viscosity.

To confirm pDNA-PKcs inhibition, 1.5*10^6^ cells were seeded in a T25 flask one day prior to irradiation. The flasks were irradiated with 8 Gy with and without inhibitor and lysates were made, 1 h after irradiation, as described above.

To determine the protein concentration in isolated protein samples a Lowry assay was performed [22]. Samples were prepared by adding loading buffer (final concentration 0.01 % bromophenol blue and 0.5 % β-mercaptoethanol). Samples were loaded on an SDS-page gel. Proteins were transferred onto an Odyssey Immobilin-P transfer membrane (Millipore). Blotting was performed for 2 h at 4 °C at 300 mA in transfer buffer (0.4 M Glycine, 5 mM Tris, 20 % Methanol). After blotting, the membranes were incubated in blocking buffer (3 % skim milk 0.05 % Tween-20 in PBS) for one hour at room temperature. Membranes were incubated with primary antibodies (Rabbit anti-vinculin (1:1000, NB129002, Abcam), rabbit anti-Phospho-DNA-PKcs S2056 (E9J4G) (1:1000, 68716S, Cell signaling Technology), mouse anti-BRCA2 (1:1000, OP95, Merck millipore)) in blocking buffer overnight at 4 °C. The membranes were washed with 0.05 % Tween-20 in PBS. After washing, membranes were incubated with secondary antibodies for 1.5 h at room temperature with the secondary antibody (1:2000, HRP anti-rabbit or HRP anti-mouse, Jackson ImmunoResearch Labs) in blocking buffer for 1–2 h at room temperature. The membrane was washed and after addition of enhanced chemiluminescence substrate (homemade) to the blots, chemiluminescence was measured with Amersham imager 600 (GE Healthcare).

## Results

### Hyperthermia radiosensitizes more to proton than to X-ray radiation

**Supplementary Fig. 1:**
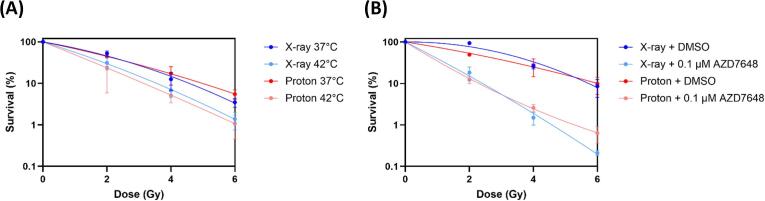
Combined plots of clonogenic survival curves. (A) Combined plot of clonogenic survivals presented in Fig. 2D. Clonogenic survival after combination treatment of either X-ray or protons with HT treatment. Error bars represent standard deviation. (B) Combined plot of clonogenic survivals presented in Fig. 3C. Clonogenic survival after combination treatment of either X-ray or protons with AZD7648. Error bars represent standard deviation.

To investigate the importance of homologous recombination in repairing proton-induced DSBs we subjected FaDu cells to SOBP proton and X-ray radiation in combination with either hyperthermia (42 °C for 1 h) or normothermia (37 °C) treatment (Fig. 2). As shown before, hyperthermia treatment indeed induces BRCA2 degradation (Fig. 2A). Additionally, the number of Rad51 foci per nucleus was determined 1 and 2 h after hyperthermia and radiation treatment to demonstrate that hyperthermia induces homologous recombination deficiency. Hyperthermia treatment leads to a loss of Rad51 focus formation (Fig. 2B and C), with this effect persisting for at least 2 h after irradiation. Together, this indicates that hyperthermia successfully induces homologous recombination deficiency. Clonogenic survival assays show that hyperthermia radiosensitizes FaDu cells to both proton and X-ray radiation (Fig. 2D). A combined plot of the X-ray and proton survival curves can be found in Supplementary Fig. 1A. A direct comparison between the X-ray and proton clonogenic survival was not made, as differences in location, and utilizing separate labs and incubators, can introduce variability between the X-ray and proton survival curves. Instead we focus on the induced radiosensitization between the monotherapy and the combination therapy with hyperthermia. The sensitization ratio (SR), defined as the D10 of monotherapy treated cells divided by the D10 of combination treated cells, was determined for both combination treatments (Fig. 2E). While the radiosensitization is modest in combination with photon radiation (SR = 1.32), hyperthermia combined with proton radiation shows a significantly stronger radiosensitizing effect (SR = 1.53). These results align with our hypothesis that cells rely more on homologous recombination after proton irradiation than after X-ray irradiation.

### Inactivation of NHEJ by inhibition of DNA-PKcs radiosensitizes equally to X-ray and to proton radiation

**Fig. 3:**
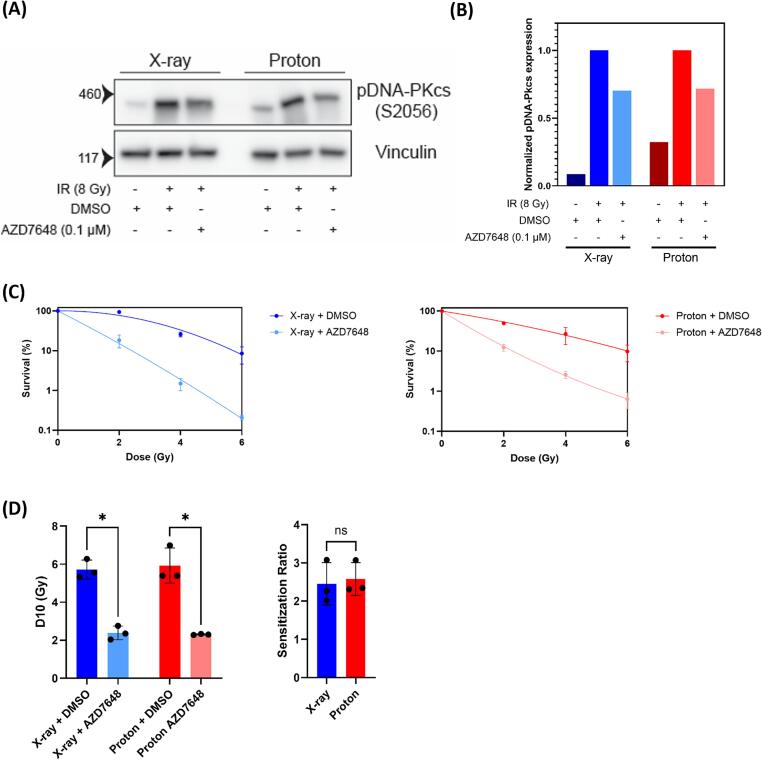
Inactivation of NHEJ radiosensitizes equally to photon and proton radiation. (A) Western blot to confirm inhibition of the phosphorylation of DNA-PKcs upon treatment with inhibitor. Vinculin was used as a loading control. (B) Quantification of phospho-DNA-PKcs expression. Expression was first corrected using loading control and then normalized to the expression of the 8 Gy DMSO sample. (C) Clonogenic survival after combination treatment of either X-ray or protons with AZD7648. Error bars represent standard deviation. (D) D10 (left) and SR (right) values obtained from the clonogenic survival after combination treatment of either X-ray or protons with AZD7648. Each black dot represents an independent experiment. Error bars represent standard deviation.

To study the role of another important DSB repair pathway, NHEJ, in the repair of proton induced DSBs, we also determined the clonogenic survival of FaDu cells after inactivation of NHEJ. NHEJ was inactivated by pharmacological inhibition of DNA-PKcs, an important player in NHEJ, using AZD7648 [4], [23]. Inhibition of DNA-PKcs was confirmed by reduced auto-phosphorylation of DNA-PKcs (ser2056) (Fig. 3A and B). Clonogenic survival assays show that NHEJ inactivation induces strong radiosensitization in combination with both X-ray and proton radiation (Fig. 3C and Supp. Fig. 1B). Unlike the hyperthermia-induced radiosensitization, there is no significant difference between the sensitization observed after proton (SR = 2.58) and photon (SR = 2.45) radiation (Fig. 3D). This indicates that, while NHEJ plays a major role in the repair of both types of radiation, there is no differential effect between the two radiation types.

### Proton radiation induces more Rad51 foci compared to X-ray radiation

**Fig. 4:**
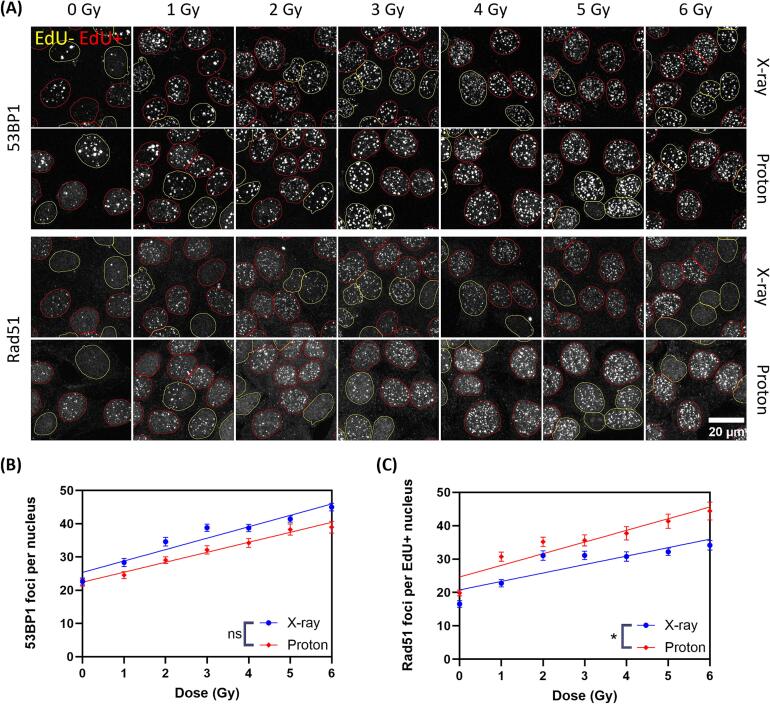
Proton radiation induces more and bigger Rad51 foci. (A) Representative images of Rad51 immunofluorescent staining 4 h after a dose gradient (0–6 Gy) of either X-ray or proton radiation. (B) Quantification of the number of 53BP1 foci per nucleus. (C) Quantification of the number of Rad51 foci per EdU positive nucleus.

To corroborate the result that hyperthermia-induced homologous recombination deficiency radiosensitizes FaDu cells more to protons than to X-rays, the direct involvement of homologous recombination was investigated further. This was done by studying Rad51 focus formation 4 h after a dose gradient (0–6 Gy) of proton and X-ray radiation (Fig. 4A). We quantified the amount of Rad51 foci per S-phase nucleus, indicated by EdU staining, as well as the number of 53BP1 foci per nucleus. To reduce the effect of the biological variability, a line was fitted through the datapoints with simple linear regression. There is no significant difference in the number of 53BP1 foci induced per Gy between proton and X-ray radiation, indicating that the total damage load is the same in both conditions (Fig. 4B). However, there is an increase in the number of Rad51 foci induced by proton radiation compared to X-ray radiation, especially at higher doses (>4 Gy) (Fig. 4C). The slopes of the fitted lines differ significantly between X-ray and proton radiation, with a slope of 2.53 and 3.49 Rad51 foci/Gy respectively. The ratio of these slopes (slope proton/slope proton = 1.38) is bigger than what would be expected solely based on the difference in relative biological effectiveness, further confirming that this is not due to a difference in total damage load. Together this shows that while the number of breaks does not differ significantly, Rad51 is recruited to more of the breaks, indicating that proton-induced DSBs are more often repaired by homologous recombination than X-ray induced breaks.

## Discussion

We have investigated the differential response in a head and neck cancer cell line after proton and photon radiation combined with hyperthermia or DNA-PKcs inhibition. We observed an increased radiosensitivity when proton radiation was combined with hyperthermia compared to the combination with photon radiation. Hyperthermia induces BRCA2 degradation and hereby homologous recombination deficiency. Furthermore, we observe an increased Rad51 focus accumulation at proton-induced DSBs compared to photon-induced DSBs, while there is no difference in the number of 53BP1 foci. Therefore, our results indicate an increased dependence on homologous recombination to repair proton-induced DNA damage than photon-induced DNA damage. When DNA-PKcs is inhibited we see an equally strong radiosensitization effect after photon and proton radiation, but no differential response, indicating the importance of NHEJ after both radiation types.

To the best of our knowledge, these are the first results showing an increased Rad51 recruitment to proton-induced DNA damage compared to photon-induced DNA damage. Depletion of Rad51 has been shown to sensitize cells more to proton than to photon radiation [7], [8], however up to now an increased recruitment of Rad51 was not shown. These results directly indicate an increased involvement of homologous recombination to repair proton-induced DSBs.

It is challenging to compare our findings to other literature due to many differences in for instance, cell type, the method of inactivating a repair pathway, and physical parameters of the proton radiation used. Most literature agrees with our findings that cells rely more on homologous recombination to repair proton than photon-induced DSBs [7], [8], although some only observe this for higher LET protons [9]. Others only observe an increased dependence on homologous recombination after carbon ion radiation, which has an even higher LET [13], [24]. Most published research does show a strong radiosensitization for NHEJ inhibition, regardless of radiation type, which is in agreement with our results [11], [12].

Most studies used non-isogenic DNA repair deficient and proficient cell lines to study DSB repair mechanisms after proton and photon irradiation. However, differences in genetic background can overshadow the marginal differences in photon and proton response induced by homologous recombination or NHEJ deficiencies [25]. It is therefore important to carefully design these types of experiments. Our approach where we make use of one head and neck squamous cell carcinoma cell line which is then subjected to either NHEJ or homologous recombination inhibition allows us to study the differential DNA damage response without the additional variability that comes with the use of non-isogenic cell lines.

The use of a DNA-PKcs inhibitor also has its flaws, which need to be acknowledged. In contrast to a full DNA-PKcs knock-out where no protein is present, inhibition of DNA-PKcs auto-phosphorylation might have additional effects. Auto-phosphorylation of DNA-PKcs is required for the dissociation of the DNA-PK complex [26], [27], [28]. Inhibition of DNA-PKcs activity can hereby result in stable DNA-PK binding to the DSB ends, preventing further processing of the breaks by other pathways [29]. This is different from the situation where there is no protein present at all. On the other hand, the use of AZD7648, which has been shown to be a potent and selective inhibitor of DNA-PKcs activity [30], does allow us to only have inhibition of DNA-PKcs upon radiation treatment and not during regular cell culture, whereas knock-out of NHEJ factors generally leads to poor fitness of cells.

Mild hyperthermia (42 °C) is a potent inducer of homologous recombination deficiency [16]. Temperatures above 42 °C, while capable of reducing BRCA2 levels, were shown to introduce additional complications [31]. For example, at 43 °C or higher, BRCA2 degradation continued, but the protein began to shift into an insoluble fraction, indicating aggregation rather than functional degradation. Furthermore, the higher temperatures resulted in the formation of aberrant RAD51 foci patterns which are associated with stalled replication forks, suggesting that excessive heat disrupts other essential processes in the S-phase of the cell cycle. Hyperthermia could also have a more general effect on DNA repair, which is not well understood [32], [33]. It has been shown that hyperthermia has an inhibitory effect on DNA-PKcs activity [32], [34]. However, this was performed with a different hyperthermia regime of 44 °C, which not only induces homologous recombination deficiency, but also additional effects [31]. Additionally, a radiosensitizing effect is frequently observed when WT or NHEJ-deficient cells are irradiated in combination with a hyperthermia regimen of 42 °C for 1 h [34], [35], whereas there is no additional effect when homologous recombination deficient cells are treated with hyperthermia [16], [35]. In summary, 42 °C provides a targeted inhibition of homologous recombination by degrading BRCA2 with limited off-target effects, while temperatures above this threshold introduce additional stress responses and protein aggregation that could compromise the effectiveness of the treatment.

The obtained results are clinically relevant, especially because of the use of hyperthermia, which is already part of clinical practice [32]. The impact of hyperthermia was also demonstrated in four clinical trials for treatment of head and neck cancer [36]. Continuous development of hyperthermia applicators has improved heating of deep-seated tumors and control of thermal dose [37]. Using hyperthermia, it is possible to non-invasively and locally induce homologous recombination deficiency. Hereby, it extends the benefits of homologous recombination deficiency status in proton treatment beyond tumors that have genetic defects in the homologous recombination pathway. Before the obtained results can be translated to the clinic, careful *in vivo* testing should be considered. *In vivo*, mild-hyperthermia also has other effects besides altering DNA repair, which was summarized in the six hallmarks of hyperthermia [38]. For instance, it improves the tumor oxygenation by altering blood flow and increasing vessel perfusion [14], [32]. Further preclinical testing is required, but our results are promising, and could provide a rational basis for the radiosensitization of proton therapy by hyperthermia.

The combination of proton therapy with hyperthermia has been investigated in various studies. Using chordoma cell lines, it was shown that combination of hyperthermia with proton therapy induces lower cell survival than proton therapy alone [39]. A similar radiosensitizing effect was observed when A549 cells were treated with the combination of proton therapy and hyperthermia [40]. In a phase I/II study on unresectable and recurrent soft tissue sarcoma the combination of proton therapy with hyperthermia has shown the safety and feasibility of the combination therapy with nearly total tumor control [41]. The combination of low LET radiation with hyperthermia has also been suggested as an alternative to high LET radiation, such as carbon ion therapy [42], [43]. Carbon ion therapy has various benefits over proton therapy such as a higher relative biological effectiveness and a reduced oxygen enhancement ratio, which could facilitate the treatment of hypoxic tumors, albeit at a much higher cost than proton therapy [43]. The combination therapy of proton radiation with hyperthermia shares many of the benefits of carbon ion therapy and could result in a mix of the advantages of proton therapy, an improved physical dose distribution, and an improved radiobiological effect similar to high LET carbon ions [43].

## CRediT authorship contribution statement

**Tim Heemskerk:** Conceptualization, Methodology, Investigation, Writing – original draft, Writing – review & editing. **Gerarda van de Kamp:** Conceptualization, Methodology, Investigation, Writing – original draft, Writing – review & editing. **Marta Rovituso:** Methodology, Writing – review & editing. **Roland Kanaar:** Conceptualization, Supervision, Funding acquisition, Writing – review & editing. **Jeroen Essers:** Conceptualization, Supervision, Funding acquisition, Writing – review & editing.

## Funding

This work was supported by the Dutch Cancer Society (INTOPROT, 12092/2018) and Varian (PROTON-DDR, 2019020), and is part of the Oncode Institute, which was partly financed by the Dutch Cancer Society.

## Declaration of competing interest

The authors declare that they have no known competing financial interests or personal relationships that could have appeared to influence the work reported in this paper.
